# Inequality in treatment use among elderly patients with acute myocardial infarction: USA, Belgium and Quebec

**DOI:** 10.1186/1472-6963-9-130

**Published:** 2009-07-30

**Authors:** Julian Perelman, Amir Shmueli, Kathryn M McDonald, Louise Pilote, Olga Saynina, Marie-Christine Closon, TECH Investigators

**Affiliations:** 1 Escola Nacional de Saúde Pública, Universidade Nova de Lisboa and CIESP, Universidade Nova de Lisboa, Avenida Padre Cruz, 1600-560 Lisbon, Portugal; 2 School of Public Health, the Hebrew University, Jerusalem, Israel; 3 Stanford University School of Medicine, Center for Primary Care and Outcomes Research, Stanford, USA; 4 Research Institute of the McGill University Health Centre, McGill University, Montreal, Canada; 5 Stanford University School of Medicine, Center for Primary Care and Outcomes Research, Stanford, USA; 6 Inter-disciplinary Center in Health Economics, School of Public Health, Université Catholique de Louvain, Belgium

## Abstract

**Background:**

Previous research has provided evidence that socioeconomic status has an impact on invasive treatments use after acute myocardial infarction. In this paper, we compare the socioeconomic inequality in the use of high-technology diagnosis and treatment after acute myocardial infarction between the US, Quebec and Belgium paying special attention to financial incentives and regulations as explanatory factors.

**Methods:**

We examined hospital-discharge abstracts for all patients older than 65 who were admitted to hospitals during the 1993–1998 period in the US, Quebec and Belgium with a primary diagnosis of acute myocardial infarction. Patients' income data were imputed from the median incomes of their residential area. For each country, we compared the risk-adjusted probability of undergoing each procedure between socioeconomic categories measured by the patient's area median income.

**Results:**

Our findings indicate that income-related inequality exists in the use of high-technology treatment and diagnosis techniques that is not justified by differences in patients' health characteristics. Those inequalities are largely explained, in the US and Quebec, by inequalities in distances to hospitals with on-site cardiac facilities. However, in both Belgium and the US, inequalities persist among patients admitted to hospitals with on-site cardiac facilities, rejecting the hospital location effect as the single explanation for inequalities. Meanwhile, inequality levels diverge across countries (higher in the US and in Belgium, extremely low in Quebec).

**Conclusion:**

The findings support the hypothesis that income-related inequality in treatment for AMI exists and is likely to be affected by a country's system of health care.

## Background

Previous research has provided evidence that socioeconomic status (SES) has an impact on invasive treatments use for acute myocardial infarction [1-7]. The causes for such inequalities remain difficult to discern. Some have argued that underprivileged patients could be more reluctant to undergo invasive procedures [6]; other evidence suggests that physicians perceive higher severity and greater post-intervention risk for low-income patients [4]; some explain disparities as the result of unfounded physicians' prejudices against specific groups such as women and black patients [8]; finally, over-provision to the better-off has also been mentioned, as a significant proportion of referrals from general practitioners to specialists are made upon patients' requests [9,10].

Focus has essentially been directed at physicians' and patients' beliefs and attitudes. Nevertheless, physicians and patients act in the framework of health care systems, whose influence on physicians' and patients' behaviors, through regulations and incentives, has been well documented in the area of cardio-vascular treatments. Recently, research has emphasized the importance of health care systems on the adoption and diffusion of high-technology treatments for cardio-vascular disease [11,12]. A recent contribution suggests that universal coverage explains most of the very low inequality in access to invasive procedures in Canada [13].

The health care organization is likely to influence not only how care is provided, but also to whom it is provided. In this study, we compare health inequality in the use of diagnosis (cardiac catheterization, CATH) and treatment (coronary artery bypass graft, CABG, and percutaneous coronary intervention, PCI) for acute myocardial infarction (AMI) between three very different health care financing systems: the US (Medicare), Quebec and Belgium. This cross-country comparison aims to elucidate how health care systems may also be a determinant of inequalities in treatment.

## Methods

### Sample

Our sample includes all patients admitted to a hospital with a diagnosis of acute myocardial infarction (AMI, code 410 of the *International Classification of Diseases, 9^th^revision, Clinical Modification: ICD-9-CM*).

We follow the exclusion criteria used in other studies [5,11,14]. We exclude patients discharged alive after a stay of less than three days, those transferred from another acute care facility to avoid double counting and those hospitalized with an AMI in the year before the index admission and patients older than 99 in order to limit variations in severity.

Our sample is restricted to all patients 65 and older hospitalized from 1993–1998. US data includes all Medicare AMI patients (1,614,922 discharges), data from Quebec and Belgium includes all AMI patients aged 65 and older (37,190 in Quebec, 49,445 in Belgium).

### Modeling diagnosis and treatment for AMI

We assume that the hospital's clinical team, when deciding treatment for AMI, is confronted with four independent alternatives. The first option is to perform neither a CATH nor any invasive treatment. The second option is for a physician to perform a CATH, but not to follow it with any invasive procedure. The third option is to perform a PCI following the CATH. The final alternative is to perform a CABG after performing either a PCI or a CATH or both.

We construct a treatment variable as a categorical dependent variable whose categories are these four alternatives. We model the probability that the patient will fall into any of those categories, using a multinomial logit model. Coefficients from multinomial logit models are difficult to interpret and potentially misleading; to overcome this difficulty, we compute the adjusted probabilities associated with each outcome holding constant all other characteristics. SAS (version 8.2) statistical package was used.

### Explanatory variables

Table 1 displays the explanatory variables included in the regressions, with their definitions. These variables are commonly used when modeling treatment choice for AMI [14]. Year dummies are included to control for technological diffusion, which may be correlated with income distribution.

**Table 1:** Explanatory variables used in the multivariate analysis

*VARIABLE*	*DEFINITION*
*Patient-related clinical indicators*	
	
FEMALE	Dummy variable taking the value '1' for female patients
AGEGRP0-AGEGRP5	One dummy variable for each age group (where AGEGRP0 includes patients aged 65–69 and AGEGRP5 patients older than 90)
YR93-YR98	One dummy variable for each year
DIST_CATH	Distance from the patient's area geographical center to the area geographical center of the nearest hospital offering cardiac CATH.
DIST_CARD	Distance from the patient's area geographical center to the area geographical center of the nearest hospital offering CABG or PCI.
COCHF	Congestive heart failure
CARD	Cardiac dysrhythmias
PULED	Pulmonary edema
SHOCK	Shock
CRF	Chronic renal failure
ARF	Acute renal failure
DIABET	Diabetes
MAL	Malignancy
CERVAS	Cerebrovascular disease
	
*Area-based socioeconomic indicators*	
	
INC	Median family income in ZIP code area of residence.
INC_Q1	The patient's area belongs to the lowest median income quintile in country X
INC_Q2	The patient's area belongs to the 2^nd ^median income quintile in country X
INC_Q3	The patient's area belongs to the 3^rd ^median income quintile in country X
INC_Q4	The patient's area belongs to the 4^th ^median income quintile in country X
INC_Q5	The patient's area belongs to the highest median income quintile in country X

We use the distance to the closest hospital with on-site cardiac facilities as the explanatory variable, given its known influence on the use of cardiac services [15]. We use straight-line distances between the patient's area geographical center and the nearest equipped hospital's area geographical center. The use of a straight-line instead of aerial distances is quite common in the literature, as studies generally assume that both measures are correlated [15,16]. Using the distance to the closest equipped hospital instead of the distance to the actual hospital used for care avoids a confounding effect; indeed, the hospital choice often determines the treatment received [17].

We use the median income of the patient's residential area in order to determine the socioeconomic status, as a typical approach [2,5]. Median incomes are not comparable across countries because of different purchasing power and definition. **US **area-based information is issued from the 1990 census. The median income is based on the taxable household income, including many sources of revenue (wages and salary, social benefits, dividends, etc.). Area-based income data for **Quebec **are issued from the Profile of Forward Sortation Areas, obtained from the 1996 Canada Census data. Median income corresponds to the median personal net income and also includes many other sources of revenue. For **Belgium**, area-based data are issued from the annual fiscal register (1995 data used for this study). Median income corresponds to the median taxable income by declaration and includes all taxable revenue. None of the countries standardize median income by the household size. Instead of using the median income itself, we distribute areas in quintiles according to their median income, providing a comparable relative measure of SES.

Adjusted probabilities of each intervention are calculated for each income quintile, controlling for age and comorbidities. Using 95% confidence intervals, we observe whether adjusted probabilities significantly differ across income quintiles. We assume that patient's median income and intervention are significantly associated whenever adjusted probabilities significantly differ between at least two income quintiles.

### Adjusting for distances and existence of on-site facilities

The *First model *does not include distances, while the *Second model *does. These separate models allow for measuring the importance of distances in explaining inequalities in use. In a *Third model*, we check whether inequality exists among patients admitted at hospitals with on-site cardiac facilities (for catheterization and revascularization). Indeed, socio-economic status may still lead to treatment disparities when the distance factor and the hospital choice are neutralized. Therefore, we use the sub-sample of those patients admitted at hospitals with on-site facilities, including 782,715 discharges in the US, 5,859 in Quebec and 14,952 in Belgium.

## Results

Patients' characteristics are displayed in Table 2. In all three countries, we observe relatively higher rates of use of cardiac procedures among patients who come from the highest-income areas. However, no income gradient is present as rates are higher in the lowest-income areas compared with the middle-income ones. Area median income and distances to hospitals with on-site cardiac facilities follow similar patterns. In the US, patients from the poorest areas live on average 45 km away from the closest hospital with on-site cardiac facilities, but patients from the richest areas live on average only 13 km away. In Quebec and Belgium, we observe an inverse U-shaped curve when comparing SES and distance from a cardiac facility. Note also that the poorest areas, situated farther from equipped facilities, are also the less populated in the US. It seems thus that, contrary to Quebec and Belgium, income and population density (a proxy of urbanization) are correlated in the US.

**Table 2:** AMI population characteristics by income quintile (unadjusted)

	*INCOME QUINTILE*				
	Q1	Q2	Q3	Q4	Q5
**USA**					
					
% CATH	32.1	31.1	31.8	34.5	35.1
% PCI	12.0	11.3	11.6	12.7	13.1
% CABG	0.7	0.7	0.7	0.8	0.8
Average distance to CATH*	58.4	54.2	43.5	27.6	16.2
Average distance to REVASC*	45.2	48.0	37.4	21.1	13.2
% equipped hospitals	51.9	44.6	45.2	51.0	51.1
% total population	10.35	13.84	17.53	24.68	33.60
					
**Quebec**					
					
% CATH	19.2	16.7	17.0	22.2	24.0
% PCI	6.7	5.6	5.9	7.0	7.6
% CABG	0.8	0.6	0.7	0.9	0.8
Average distance to REVASC*	55.5	80.8	88.6	41.7	24.7
% equipped hospitals	19.6	9.9	15.4	19.8	18.9
% total population	21.16	26.54	20.62	17.89	13.78
					
**Belgium**					
					
% CATH	21.6	14.2	13.0	17.3	22.0
% PCI	5.8	3.4	3.0	5.1	7.2
% CABG	1.6	0.7	1.0	1.0	1.5
Average distance to CATH*	19.8	32.4	33.8	34.5	26.1
Average distance to REVASC*	13.4	14.4	14.7	15.1	11.3
% equipped hospitals	39.1	24.1	22.8	29.9	39.2
% total population	23.95	22.81	18.82	17.35	17.46

Note: *Average distance is expressed in kilometers (1 mile = 1.67 km). Average distance to CATH measures the average distance to the nearest hospital with on-site facilities for CATH ONLY. Average distance to REVASC measures the average distance to the nearest hospital with on-site facilities to perform PCI or CABG.

Adjusted probabilities appear in Table 3. The value denoted 'range' gives the percentage difference between the categories exhibiting the lowest and the highest adjusted probability. We present the findings by country.

**Table 3:** Adjusted probabilities for income quintiles

*USA*	*CATH*	*PCI*	*CABG*	*No treatment*
**Procedure use in all hospitals, unadjusted for distance (Model 1)**. N = 1,614,922				
INC_Q1	0.1374*	0.1085*	0.0725*	0.6816
INC_Q2	0.1372*	0.1031*	0.0710*	0.6887
INC_Q3	0.1442*	0.1059*	0.0700*	0.6799
INC_Q4	0.1538*	0.1138*	0.0741*	0.6583
INC_Q5	0.1564*	0.1172*	0.0758*	0.6506
Range	13.99%	13.68%	8.29%	5.86%
**Procedure use in all hospitals, adjusted for distance (Model 2)**. N = 1,614,992				
INC_Q1	0.1600*	0.1168*	0.0766*	0.6466
INC_Q2	0.1573*	0.1149*	0.0766*	0.6512
INC_Q3	0.1584*	0.1127*	0.0730*	0.6559
INC_Q4	0.1592*	0.1149*	0.0747*	0.6512
INC_Q5	0.1564*	0.1172*	0.0756*	0.6508
Range	1.72%	3.99%	4.93%	1.44%
**Procedure use in hospitals that provide on-site CABG and PCI (Model 3)**. N = 782,715				
INC_Q1	0.1932*	0.1987*	0.1299*	0.4782
INC_Q2	0.1924*	0.2125*	0.1422*	0.4529
INC_Q3	0.1940*	0.2244*	0.1458*	0.4358
INC_Q4	0.1900*	0.2292*	0.1496*	0.4312
INC_Q5	0.1889*	0.2395*	0.1549*	0.4167
Range	2.70%	20.53%	19.25%	14.76%

*QUEBEC*	*CTH*	*PCI*	*CABG*	*No treatment*

**Procedure use in all hospitals, unadjusted for distance (Model 1)**. N = 37,190				
INC_Q1	0.0899*	0.0533*	0.0076	0.8492
INC_Q2	0.0834*	0.0474*	0.0061	0.8631
INC_Q3	0.0753*	0.0458*	0.0066	0.8723
INC_Q4	0.0980*	0.0521*	0.0074	0.8425
INC_Q5	0.1113*	0.0578*	0.0077	0.8232
Range	47.81%	26.20%	26.23%	5.96%
**Procedure use in all hospitals, adjusted for distance (Model 2)**. N = 37,190				
INC_Q1	0.0964*	0.0571*	0.0085	0.8380
INC_Q2	0.0925*	0.0538*	0.0077	0.8460
INC_Q3	0.0838*	0.0525*	0.0080	0.8557
INC_Q4	0.1011*	0.0550*	0.0078	0.8361
INC_Q5	0.1113*	0.0578*	0.0077	0.8232
Range	32.82%	10.10%	10.39%	3.95%
**Procedure use in hospitals that provide on-site CABG and PCI (Model 3)**. N = 5,859				
INC_Q1	0.1312	0.1448	0.0396	0.6844
INC_Q2	0.1274	0.1434	0.0446	0.6846
INC_Q3	0.1322	0.1302	0.0283	0.7093
INC_Q4	0.1462	0.1333	0.0390	0.6815
INC_Q5	0.1367	0.1562	0.0338	0.6733
range	14.76%	19.97%	57.60%	5.35%

*BELGIUM*	*CATH*	*PCI*	*CABG*	*No treatment*

**Procedure use in all hospitals, unadjusted for distance (Model 1)**. N = 49,445				
INC_Q1	0.1261*	0.0383*	0.0111*	0.8245
INC_Q2	0.0942*	0.0255*	0.0061*	0.8742
INC_Q3	0.0836*	0.0227*	0.0082*	0.8855
INC_Q4	0.0998*	0.0346*	0.0078*	0.8578
INC_Q5	0.1191*	0.0458*	0.0115*	0.8236
range	26.35%	101.76%	88.52%	7.40%
**Procedure use in all hospitals, adjusted for distance (Model 2)**. N = 49,445				
INC_Q1	0.1153*	0.0363*	0.0100*	0.8384
INC_Q2	0.1090*	0.0281*	0.0073*	0.8556
INC_Q3	0.0992*	0.0260*	0.0099*	0.8649
INC_Q4	0.1202*	0.0405*	0.0097*	0.8296
INC_Q5	0.1191*	0.0458*	0.0115*	0.8236
range	21.17%	76.15%	57.53%	5.01%
**Procedure use in hospitals that provide on-site CABG and PCI (Model 3)**. N = 14,952				
INC_Q1	0.2669*	0.1293*	0.0372*	0.5666
INC_Q2	0.2633*	0.1127*	0.0299*	0.5941
INC_Q3	0.2753*	0.1042*	0.0419*	0.5786
INC_Q4	0.2853*	0.1329*	0.0331*	0.5487
INC_Q5	0.2701*	0.1511*	0.0378*	0.5410
Range	8.36%	45.01%	40.13%	9.81%

Notes: estimates for SES variables obtained from multinomial logit regression (adjusted probabilities are adjusted for age groups, year and comorbidities). This Table should be read as follows: e.g., for individuals from the US living in the lowest quintile (Q1) income area, the adjusted probability of undergoing a CATH is 0.1374, controlling for age, sex, comorbidities and year. The 'no treatment' category is obtained by simple subtraction; hence it does not include any indication about statistical significance.

*Estimate significantly differs from at least one other quintile's estimate at p ≤ 0.05.

### United States

#### Procedure use in all hospitals, unadjusted for distance (Model 1)

In the US, patients from highest-income areas have a significantly higher adjusted rate of procedures followed by patients living in Q4-areas. Patients from lowest-income areas (Quintile 1, referred to as Q1) exhibit higher rates than patients in Q2-areas for CATH, than Q2- and Q3-areas for PCI and CABG. Patients from the poorest areas have a 68.2%-adjusted probability of not receiving any treatment, compared with a 65.1%-adjusted probability among patients from richest areas.

#### Procedure use in all hospitals, adjusted for distance (Model 2)

Results change dramatically when distance is taken into account. Adjusted probabilities significantly increase for patients from the poorest areas (from 13.7 to 15.6% for CATH). Consequently, even though differences among groups are still significant, they become rather weak in magnitude. The discrepancy between the lowest and the highest adjusted rate decreases from 13.99 to 1.72% for CATH, 13.68 to 3.99% for PCI.

#### Procedure use in hospitals that provide on-site CABG and PCI (Model 3)

We observe a clear income gradient for invasive interventions. For PCI, the adjusted rate is 19.9% for patients from the poorest areas compared with 23.9% for patients from richest areas, with a consistent positive correlation between level of income and rate of treatment. For bypass surgery, respective rates are 13 and 15.5%. Patients from the poorest areas have a 47.8%-adjusted probability of receiving no treatment at equipped hospitals versus 41.7% among patients from the richest areas. On the contrary, differences in adjusted probabilities remain quite weak in magnitude, although significant, for CATH (2.70% discrepancy between the lowest and the highest adjusted rate).

To summarize, the highest rates of diagnosis and intervention are found among patients from the richest areas. However, as distances are included, differences across income categories decline yet remain significant. Nevertheless, we observe a clear income gradient among patients admitted to hospitals with on-site cardiac facilities.

### Quebec

#### Procedure use in all hospitals, unadjusted for distance (Model 1)

For all treatments, the highest adjusted rates are found among patients from the richest areas followed by patients in Q4- and Q1-areas. Like in the US, patients from the poorest areas do not exhibit the lowest rates of treatment. Nevertheless, adjusted probabilities do not significantly differ in the case of CABG. The highest income group exhibits the lowest rate of 'no treatment' (82.3%).

#### Procedure use in all hospitals, adjusted for distance (Model 2)

When controlling for distances, differences across income categories become somewhat weak in magnitude for PCI: the difference between extreme values decreases from 26 to 10%. However, for CATH, discrepancies remain high between extreme categories of SES (32%). Differences remain non-significant for CABG. For CATH and PCI, the distribution remains similar, i.e., we observe a J-shaped curve with the highest rates among patients from the richest areas followed by those from the poorest ones.

In the 'no treatment' case, patients from richest areas still exhibit the lowest adjusted rates. This outcome confirms that the higher adjusted rate for CATH among extreme categories is the sign of a 'better' treatment. Indeed, higher rates in the category 'CATH only' could be interpreted as a less intensive treatment, that is, a diagnosis that is not followed by an invasive procedure. This is not the case.

#### Procedure use in hospitals that provide on-site CABG and PCI (Model 3)

Income plays no role among patients admitted to hospitals with on-site cardiac facilities: adjusted rates do not significantly vary across income groups.

### Belgium

#### Procedure use in all hospitals, unadjusted for distance (Model 1)

In Belgium, for the three interventions, the highest adjusted rates are found among patients who come from 'extreme' areas, i.e. poorest and richest areas (Q1 and Q5). Adjusted PCI rates are 3.83% for patients from the poorest areas, then decrease to 2.55 and 2.27% for patients in the second and third quintile and increase again to 3.46 and 4.58% for the third and fourth quintile.

When we consider patients who did not receive treatments (last column), the lowest adjusted rates are found among patients from the poorest and richest areas (82.45 and 82.36% respectively).

#### Procedure use in all hospitals, adjusted for distance (Model 2)

When distances are included, we observe a J-shaped curve for the three interventions. Distance plays a minor role in Belgium, since distances to equipped hospitals differ on average by only 3.81 km between the richest and poorest areas.

#### Procedure use in hospitals that provide on-site CABG and PCI (Model 3)

The highest rate of treatment among patients from the richest area exists only in the case of PCI. The inverse J-shaped curve remains for the 'no-treatment' case, with higher rates among patients from the third quintile (59.41%), and lowest among patients from the poorest and richest areas (56.66 and 54.10% respectively). Even when access to hospitals with on-site cardiac facilities is fully insured, inequality remains across income categories.

## Discussion

Our findings show that in all countries, the highest adjusted treatment rates are found among patients from the highest income areas, when *not accounting for distances between patient and hospital*. This is true however to a lesser extent in Quebec. In the US, distances from hospitals with on-site catheterization and revascularization facilities are shorter for richer areas. As a result, patients from those areas have greater use of high-technology interventions such as PCI or cardiac surgery.

When *distances are accounted for*, inequality becomes quite low in the US, and is also reduced in Quebec. That is, if the issue of distance is controlled for, all three health care systems achieve low levels of inequality in use of high-technology interventions. Thus, it appears that income inequality is largely related to location of hospitals with catheterization and revascularization capabilities, particularly in the US.

In the US, the richer the area, the closer the cardiac services. By contrast, greatest distances are observed in Belgium and Quebec among middle-income areas. It is thus not surprising to observe a more pronounced income gradient in the US that almost disappears when controlling for distances to equipped facilities. Hence, the main cause of income inequality does not seem to be income itself but instead distances from equipped hospitals. These findings substantiate those of previous studies showing the impact of geographic proximity of revascularization services on service utilization [15,17].

The question however remains about why income and distance to equipped hospitals are strongly related in the US while this is not the case in Belgium and Quebec. The first straightforward explanation is that the poorest areas in the US are also the least populated (rural areas), in contrast with Belgium and Quebec.

However, this double disadvantage of poverty and non-urban setting may also be related to organizational characteristics. In particular, high-technology procedures are costly in terms of equipment and human capital [12]; when hospitals are free to keep their profits and invest according to their own interests, investments will concentrate among hospitals with more financial capacity or with greater expected profitability of treatments. Poor or inadequate regulation of investments may thus explain the above findings. Consider the US, in which investments in technology are generally not regulated. In densely populated urban areas, demand for cardiac procedures is high. As a result, large hospitals use their profits to invest in medical equipment that give their patients access to high technology treatments [18]. However, rural areas in the US, which are poorer than urban areas, do not have the money to invest and thus patients must seek cardiac treatments from facilities located far away, generally in the nearest city.

Furthermore, insurance may also play a role. Studies have shown that hospital's investments in the US are driven by expected financial returns, which depend upon payer type [16]. Fee-for-service (FFS) commercial payers provide the greatest average returns, followed by Medicare, HMOs and Medicaid. Meanwhile, patient's insurance type and income are known to be related (FFS is more prevalent in high-income areas, e.g. [4]). Hence, hospitals in richer areas are more likely to invest in costly new techniques because their profitability is guaranteed by the higher payments of their patients' insurance schemes. Thus Medicare patients from richer and urban areas may benefit from greater use of high-technology procedures due to their location closer to hospitals with on-site cardiac facilities.

Among *patients admitted to hospitals with on-site cardiac facilities*, we get a classical social gradient in the US, and still some inequality in favor of the better-off in Belgium in the case of PCI. This is surprising since Belgium and US Medicare are characterized by a generous universal coverage and distances to cardiac services are less relevant at this stage. A myriad of causes may explain this finding, and our study was not designed to address them. Potential explanations include the differences in patients' willingness to undergo invasive interventions, physician prejudices, and unobserved differences in severity of disease [4,6,8-10].

Special emphasis should however be given to Quebec, which does not exhibit significant inequality within this sub-sample, contrary to Belgium and the US. It is hardly plausible that the causes mentioned already do not apply to Quebec, since universal coverage is common to the three systems. Organizational factors may once again be at play. In particular, Medicare patients in the US may be insured through HMOs or FFS, leading to different use of cardiac services [19]. In Belgium, anecdotal evidence suggests that patients with supplementary private insurance may receive a higher-quality treatment due to their higher profitability for the hospital. The greater homogeneity of the insurance system in Quebec may prevent income-related inequality to occur among patients treated at fully equipped hospitals. A study including a larger number of countries with different health care system could test these assumptions.

### Study limitations

Our results are obtained using area-based SES variables. Geronimus and Bound [20] have measured that, using area-based variables instead of individual-level ones, the impact of SES on health is likely to be underestimated due to area's socio-economic heterogeneity. For example richer patients, who can afford costly treatments, will be attributed to the low median income of their living area, resulting in underestimation of results. Areas in the US have an average population of 8,440, compared to 17,170 in Belgium and 19,037 in Quebec. Hence, heterogeneity is likely to be higher in Belgium and Quebec, partly explaining the lower impact of SES in these countries. However, other authors suggest that people's health care use is affected by area factors, regardless of the individual SES[21]; this may counteract the expected underestimation of the SES impact.

Note also that the areas' heterogeneity in distances may differ between countries. Belgian areas are too small to exhibit strong heterogeneity in distances and the Forward Sortation Areas in Quebec are either urban or rural. By contrast, US census tracks, although they are designed to produce homogenous populations, have varying land area depending on the population density. Considering that the patient lives in his area's geographical center is a limitation which likely leads to underestimate the impact of distance in the US. Note that, despite this limitation, distances are shown to be a crucial determinant in our study.

Also, the use of administrative data does not include as detailed clinical information on severity of illness as a detailed survey would. Hannan et al. [22] show that administrative data fails to distinguish co-morbidities and complications, and does not contain information on previous open-heart surgery, nor on ejection fraction. However, administrative data generally have the advantage of including more patients and representing a wider geographical area.

Finally, our sample for the US is restricted to Medicare patients, while data for Belgium and Quebec include all patients older than 65. One may argue, however, that the non-Medicare patients are marginal among the elderly (5% people). In addition, the income distribution in the US is more unequal than in Belgium and Quebec (according to the World Bank development indicators [23], the Gini index was 40.8 in the US, 32.6 and 33.0 in Canada and Belgium respectively). Hence, US Medicare patients may be more homogenous than the whole US population, but not more homogenous than the Belgian or Quebec samples. Meanwhile, restricting our sample to Medicare patients enables a comparison of health care schemes that only differ in easily identifiable peripheral characteristics. This facilitates interpretation of results and comparisons across countries.

## Conclusion

Our outcomes first clearly indicate that income-related inequality does exist in use of high-technology treatment, and that this inequality is not explained by differences in the patients' health condition. This confirms results from previous literature. Inequality is largely explained, in the US and Quebec, by location of care. When distances are accounted for, inequality substantially decreases in magnitude. However, in both Belgium and the US, inequalities persist among patients admitted to hospitals with on-site cardiac facilities, rejecting the hospital location effect as the only explanation for inequalities.

Secondly, inequality differs across countries. As stated above, some features of hospital financing may exacerbate income-related inequalities in treatment, such as insurance type and investment regulations. Due to the divergence of these features between countries, we have good reason to believe they are affecting the diagnosis and treatment of AMI patients. Those assumptions should be tested using a larger set of countries making available a larger array of health care systems.

## Competing interests

The authors declare that they have no competing interests.

## Authors' contributions

All authors have contributed to the statistical analysis, results discussion and manuscript drafting. JP was however the main person responsible for the statistical analysis and drafted the first version of the manuscript jointly with KMM and her research assistant. AS contributed more specifically to the statistical analysis and to discussing theoretical issues. OS was more involved in the data gathering and the statistical analysis. LP particularly discussed all aspects related to cardiovascular disease and its treatment. The main idea of this study was given by MCC, who launched the project, coordinated the work and helped more specifically to draft the manuscript.

This paper is part of the research undertaken by the Technological Change in Health Care (TECH) Network. The TECH group is an international collaboration of investigators that has been developing new evidence on cross-country differences in trends in treatment, resource costs and health outcomes for common health problems, with a specific focus on patients hospitalized for acute myocardial infarction. The TECH group has adopted the rule for its submissions that all authors would be listed under the denomination "and the TECH group". All TECH Group members have contributed through providing data and through commenting the work during several meetings.

## Pre-publication history

The pre-publication history for this paper can be accessed here:


